# A prospective series of acute rivaroxaban overdose, coagulopathy and bleeding complications (ATOM 11)

**DOI:** 10.1002/bcp.70588

**Published:** 2026-04-24

**Authors:** Katherine Z. Isoardi, Angela L. Chiew, Keith Harris, Geoffrey K. Isbister

**Affiliations:** ^1^ Clinical Toxicology Unit Princess Alexandra Hospital Brisbane Australia; ^2^ Queensland Poisons Information Centre Queensland Children's Hospital Brisbane Australia; ^3^ Faculty of Medicine University of Queensland Brisbane Australia; ^4^ Clinical Toxicology Unit Prince of Wales Hospital Sydney Australia; ^5^ New South Wales Poisons Information Centre Children's Hospital at Westmead Westmead Australia; ^6^ School of Medicine University of New South Wales Sydney Australia; ^7^ Department of Clinical Toxicology Calvary Mater Newcastle Hospital Newcastle Australia; ^8^ Clinical Toxicology Research Group University of Newcastle Newcastle Australia

**Keywords:** bleeding complications, coagulopathy, direct oral anticoagulants (DOACs), overdose, rivaroxaban

## Abstract

**Aim:**

There is limited research describing rivaroxaban overdose. We aim to describe rivaroxaban overdose, coagulopathy and bleeding complications.

**Methods:**

This is a prospective series of acute adult (>14 years) rivaroxaban overdose presentations >40 mg enrolled in the Australian Toxicology Monitoring study and presenting to three Australian clinical toxicology units from August 2014 to August 2024. Data collected included demographics, ingestion details, coagulation profile, rivaroxaban concentrations, significant bleeding (with haemoglobin decrement) and outcome.

**Results:**

There were 58 presentations in 52 patients, 29/52 (56%) males, median age of 49 years (range: 18–93 years). The median reported dose ingested was 200 mg (IQR: 92.5–400 mg, range: 45–1120 mg). Co‐ingestants were taken in 49 (84%), most commonly mirtazapine and paracetamol. A raised international normalized ratio (INR) of >1.5 occurred in 46 (79%) with a median peak INR of 2.5 (IQR: 1.7–3.7). The median duration for an elevated INR to return to ≤1.5 was 22 h (IQR: 14–38 h). Significant bleeding occurred in two patients; both were managed conservatively without factor replacement. Rivaroxaban concentrations were available in 11 cases. There was a strong correlation (*R* ^2^ = 0.83, *p* < .001) between rivaroxaban concentration and INR. An INR of ≤1.5 was associated with therapeutic rivaroxaban concentrations. There was one death attributed to co‐ingested amlodipine.

**Conclusion:**

Bleeding was uncommon following rivaroxaban overdose. The INR appears to be a good indicator of coagulopathy, with an INR of ≤1.5 approximating therapeutic rivaroxaban concentrations. Observation of patients following acute rivaroxaban overdose until the INR ≤ 1.5 seems reasonable.

## Graphical abstract

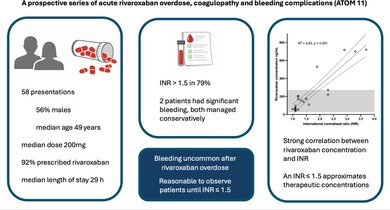

**What is already known about this subject**

- Rivaroxaban is an oral anticoagulant acting through direct factor Xa inhibition.
- There is limited literature describing rivaroxaban in overdose.

**What this study adds**

- Bleeding following rivaroxaban overdose was uncommon.
- The international normalized ratio (INR) was strongly associated with rivaroxaban concentrations, with an INR of ≤1.5 consistent with a therapeutic concentration of rivaroxaban.
- Observation following rivaroxaban overdose until an INR of ≤1.5 seems reasonable.

## 1 INTRODUCTION

Rivaroxaban is a direct factor Xa inhibitor that was first approved in Australia and the United Kingdom in 2008 for the prevention of venous thromboembolism. It has predictable pharmacokinetics and pharmacodynamics and does not need routine laboratory monitoring.^1^ Its use has increased over the last decade, overtaking warfarin to be the second most popular anticoagulant in Australia behind apixaban.^2^

There is limited research describing rivaroxaban in overdose, with the main clinical effect being anticoagulation and complication, haemorrhage. There are multiple case reports of massive overdose (dose range 1200–1960 mg), in which the patients have remained asymptomatic.^3^, ^4^, ^5^ A poison centre study of 198 rivaroxaban exposures in the United States largely described cases of therapeutic error.^6^ There were only six cases of overdose in this series, with none developing bleeding. The doses ingested were not reported. Another American poison centre series of 322 antiplatelet and anticoagulant overdoses reported 40 cases of rivaroxaban overdose, with 6/40 having bleeding of which 4/40 had major bleeding.^7^ Again, no dose information was provided, making any development of risk assessment based on dose for guidelines impossible.

With such little reported experience, the severity of coagulopathy following rivaroxaban overdose is unclear, as is the frequency of bleeding complications, the duration of observation required and the criteria for discharge.

Rivaroxaban concentrations can be measured using liquid chromatography/tandem mass spectrometry, or estimated by anti‐factor Xa activity, which may not be readily available, particularly in smaller facilities.^2^ The prothrombin time and international normalized ratio (INR) are readily available, but inter‐laboratory variation in thromboplastin reagents has led to it being considered inappropriate for therapeutic rivaroxaban monitoring.^8^ It is unclear if the prothrombin time or INR can be used to monitor anticoagulation following rivaroxaban overdose, particularly in determining the duration of observation.

We aim to describe the characteristics and time course of rivaroxaban overdoses, including the coagulopathy and bleeding complications, to better guide management.

## 2 METHODS

### 2.1 Study design, setting and participants

This prospective series is part of the Australian TOxicology Monitoring (ATOM) Study. We recruited patients from 1 August 2014 to 31 July 2024 from two Australian state poisons information centres (Queensland and New South Wales) and three clinical toxicology units (Princess Alexandra Hospital in Brisbane, Calvary Mater Newcastle Hospital and the Prince of Wales Hospital in Sydney). Adult patients (>14 years) taking an acute overdose of rivaroxaban >40 mg were included in the study. The minimum dose was determined as being more than twice the defined daily dose of rivaroxaban.

### 2.2 Data collection

A preformatted data sheet (Figure S1) was completed by the treating clinician and provided to the study team. Any missing information was supplemented from the patient medical record. Data collected included patient demographics (age, sex, prescribed rivaroxaban and indication for rivaroxaban), ingestion details (reported dose, time of ingestion and co‐ingestions), bleeding episodes, investigations (international normalized ratio [INR], rivaroxaban concentration and haemoglobin concentration), treatment (charcoal and blood products) and disposition (admitting ward and length of stay). Rivaroxaban concentrations, where available, were estimated using an anti‐factor Xa assay.

### 2.3 Analysis

Descriptive statistics were used with continuous data described by medians, interquartile ranges (IQR) and ranges. The association between the INR and rivaroxaban concentrations was assessed with the Pearson's correlation co‐efficient. All analyses and figures were performed using GraphPad Prism Version 10.6.1 for macOS.

### 2.4 Ethical considerations

The ATOM study has ethics approval from respective state ethics committees (South Eastern Sydney Local Health District Human Research Ethics Committee HREC/12/POWH/165, Children's Health Queensland Human Research Ethics Committee HREC/14/QRCH/105), and all patients recruited to the ATOM study provided consent to be included in the study. All three clinical toxicology units also have approval to include de‐identified patient data in observational research (Metro South Human Research Ethics Committee HREC/14/QPAH/308, South Eastern Sydney Local Health District Human Research Ethics Committee HREC/12/184: LNR/12/POW/355, Hunter New England Human Research Ethics Committee HREC/05/03/09/3.11).

#### 2.4.1 Nomenclature of targets and ligands

Key protein targets and ligands in this article are hyperlinked to corresponding entries in http://www.guidetopharmacology.org and are permanently archived in the Concise Guide to PHARMACOLOGY 2021/22.^9^

## 3 RESULTS

There were 58 presentations among 52 patients (male 28/52, 54%) with a median age of 49 years (range 18–93 years). Recruitment was fairly consistent over the period with a peak of 10 patients recruited in 2021 (Figure S2). In cases in which prescription data were known, rivaroxaban was a prescribed medication in 45/49 (92%). The most common indication was venous thromboembolism (*n* = 30) followed by atrial fibrillation (*n* = 14). The median reported dose of rivaroxaban ingested was 200 mg (IQR: 92.5–400 mg, range 45–1120 mg). The median time to presentation to hospital following ingestion was 3.3 h (IQR 1.6–5.4 h). Co‐ingestants were taken in 49 (84%), most commonly mirtazapine and paracetamol (Table 1). Charcoal was administered to patients in 13 (22%) presentations, at a median of 3.0 h (IQR 1.7–5.7 h) post‐ingestion.

**TABLE 1:** Baseline characteristics of 58 presentations of acute rivaroxaban overdose.

Characteristic	*n*	
Patients	52	
Male	28	54%
Median age in years (range)	49 years	(18–93 years)
Prescribed rivaroxaban^a^	45	92%
Indication		
Venous thromboembolism	30	
Atrial fibrillation	14	
Left ventricular thrombus	1	
Median dose ingested in mg (IQR)	200 mg	(92.5–400 mg)
Reported co‐ingestion	49	85%
Mirtazapine	12	21%
Paracetamol	8	14%
Atorvastatin	7	12%
Metformin	7	12%
Venlafaxine	7	12%
Diazepam	6	10%
Escitalopram	5	9%
Metoprolol	5	9%
Pantoprazole	5	9%
Quetiapine	5	9%
Multiple co‐ingestions	41	71%

^a^ Prescription data available for 49 patients.

**FIGURE 1:**
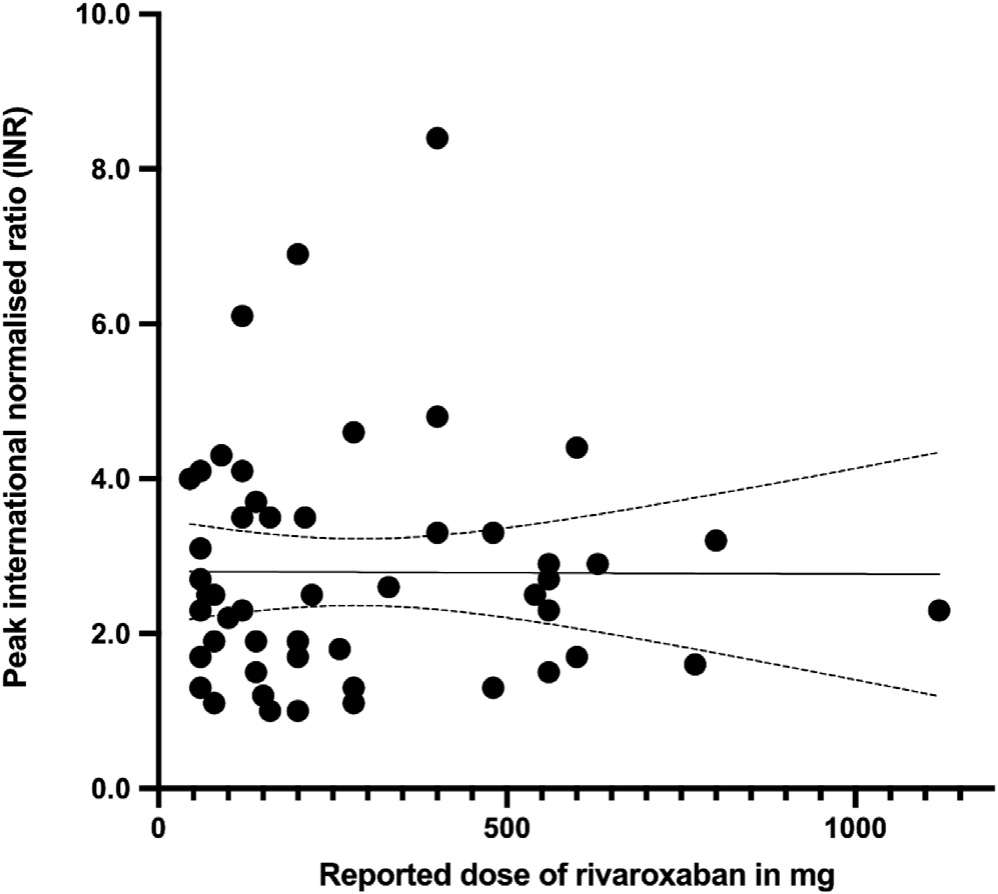
Graph of reported dose of rivaroxaban ingested and corresponding peak international normalized ratio (INR) in 58 patients taking an acute overdose of rivaroxaban. Slope suggests no association between reported dose of rivaroxaban ingested and peak INR.

**FIGURE 2:**
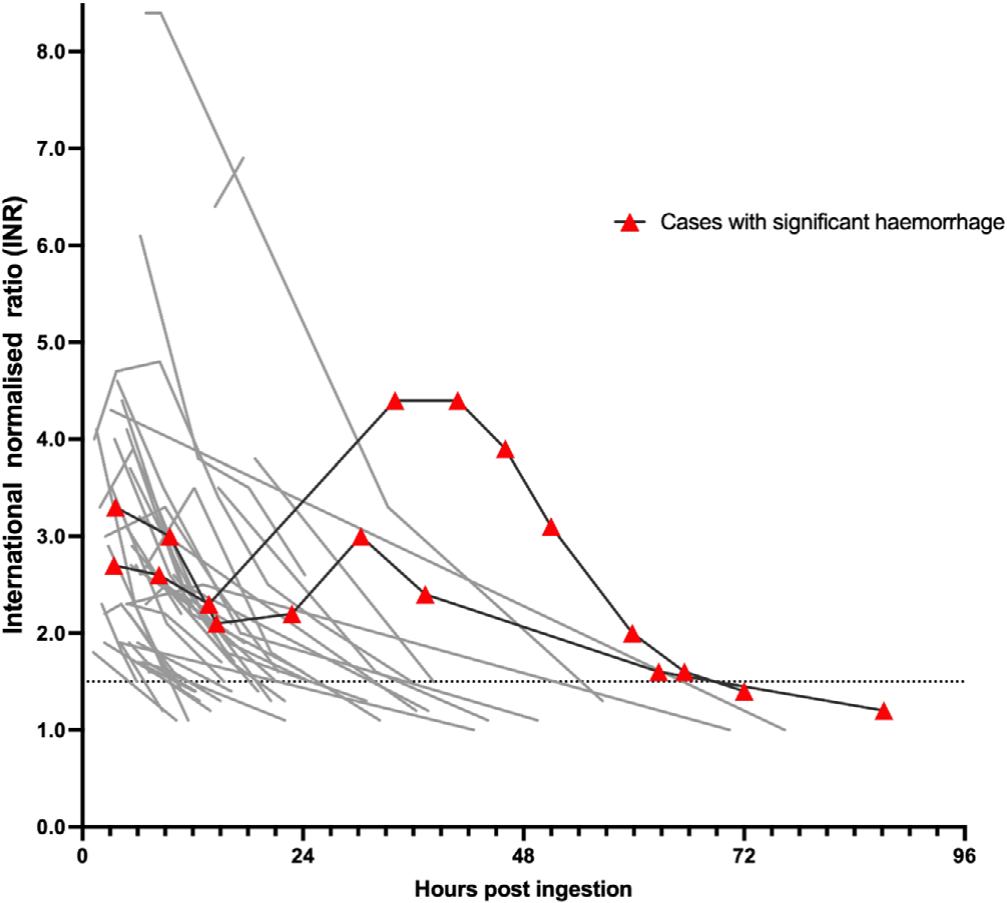
Graph plotting international normalized ratio (INR) values over time in 42 presentations of acute rivaroxaban overdose with peak INR > 1.5 and known times of ingestion. Two presentations with significant haemorrhage resulting in a fall in haemoglobin concentration are denoted with red triangular data points. The dotted horizontal line represents an INR of 1.5.

A raised international normalized ratio (INR) of >1.5 occurred in 46 (79%) with a median peak INR of 2.5 (IQR: 1.7–3.7). There was no association between reported dose ingested and peak INR (Figure 1). The median peak INR was similar in the group receiving charcoal compared to the group that did not receive charcoal (2.7 *vs*. 2.5, Figure S3). The median time to peak INR was 5.2 h (IQR: 3.5–8.7 h, range: 1.3–41 h), whereas the median duration for an elevated INR to return to ≤1.5 was 22 h (IQR: 14–38 h; range: 5.9–96 h; Figure 2).

There were five presentations with documented bleeding. Three were minor, described as either small amounts of rectal bleeding or mild haematuria, and not associated with a fall in haemoglobin. There was significant bleeding in two cases. The first was an 80‐year‐old male who ingested 600 mg rivaroxaban with 40 g paracetamol. His initial paracetamol concentration taken 3.4 h post‐concentration was 254 mg/L. He received acetylcysteine over 60 h and was admitted for observation. His peak alanine aminotransferase was 26 U/L (reference range < 45 U/L). His peak INR was 4.4, and it took 72 h for the INR to return to ≤1.5 (Figure 2). He had extensive oozing from cannula sites, for which the treating team applied pressure bandages. His haemoglobin fell from 129 g/L on arrival to hospital to 67 g/L on Day 4 following the overdose, and he received two units of packed red blood cells. He did not receive any other blood products. The second case was a 58‐year‐old male who ingested 400‐mg rivaroxaban with 500‐mg amitriptyline, 10‐g paracetamol and 600‐mg codeine. He was ventilated in the prehospital setting for an altered level of consciousness and transferred to the intensive care unit for observation. His initial paracetamol concentration taken 3.5 h post‐concentration was 114 mg/L. He received acetylcysteine over 20 h, and his peak alanine aminotransferase was 37 U/L. His peak INR was 3.3, which was ≥1.5 for 87 h (Figure 2). He developed a pulmonary haemorrhage while being mechanically ventilated. His haemoglobin dropped from 128 g/L on presentation to 81 g/L on Day 3 post‐ingestion. He was managed conservatively and did not receive blood products.

Two patients received factor replacement to treat coagulopathy. Neither patient had bleeding at the time of factor replacement. The first was a 43‐year‐old female that took 260‐mg rivaroxaban with escitalopram, quetiapine, gabapentin and mirtazapine. Her peak INR was 1.8 on presentation 1.3 h post‐ingestion. She received prothrombin complex concentrate prophylactically 5 h post‐ingestion. Her INR was ≤1.5, 10‐h post ingestion. The second patient was a 23‐year‐old female that took 70‐mg rivaroxaban with cyclizine, escitalopram, lorazepam, lansoprazole and thiamine. Her peak INR was 2.5, 13‐h post‐ingestion. She reported a small amount of PR bleeding but had no evidence of PR bleeding on examination. She received prothrombin complex concentrate, tranexamic acid and two units of fresh frozen plasma between 5 and 9 h post‐ingestion. Her INR remained ≥1.5 for 70 h.

Most presentations (*n* = 32, 55%) were managed in the emergency department or short stay treatment area, with 10 (17%) admitted to intensive care for treatment of co‐ingestants. The median length of stay was 29 h (16–79 h). There was one death attributed to vasoplegic shock due to co‐ingested amlodipine and telmisartan.

**FIGURE 3:**
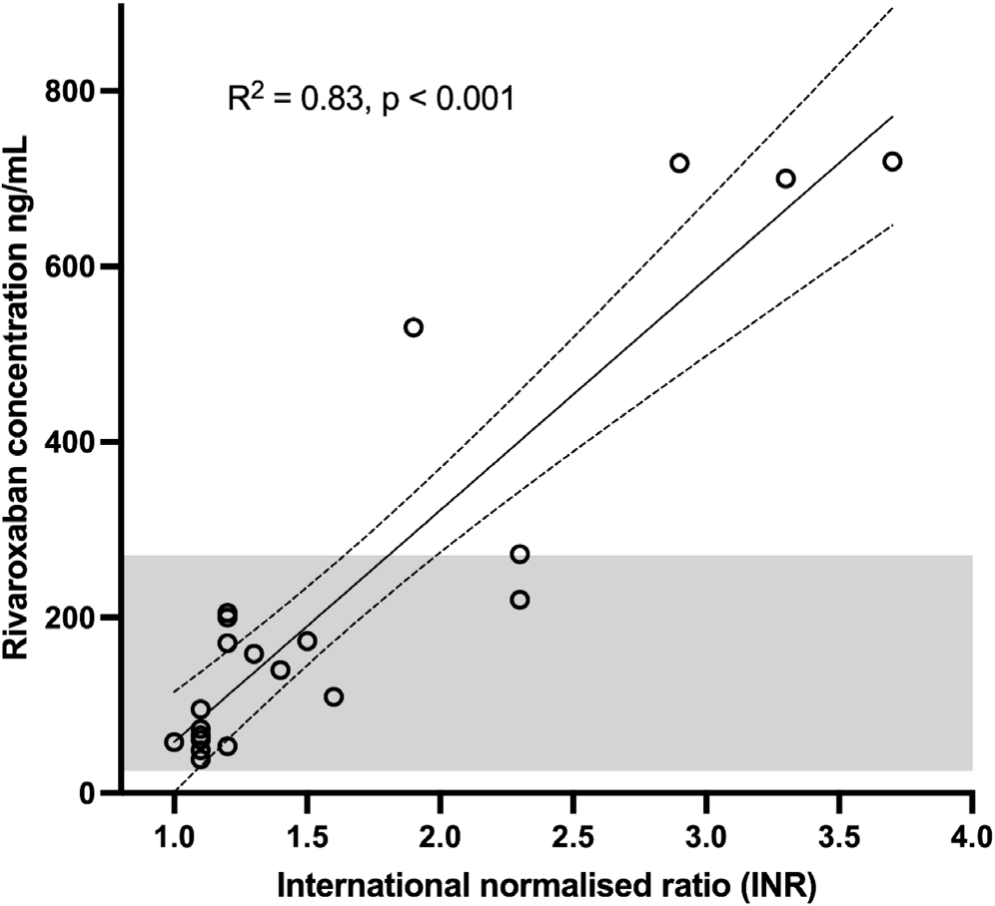
Graph of international normalized ratio (INR) and corresponding rivaroxaban concentration calculated using an anti‐factor Xa assay. The shaded area denotes the therapeutic range for rivaroxaban.

Rivaroxaban concentrations were available in 11 (19%) cases. There was a strong correlation (*R* ^2^ = 0.83, *p* < .001) between rivaroxaban concentration and INR (Figure 3). An INR of ≤1.5 was associated with therapeutic rivaroxaban concentrations.

## 4 DISCUSSION

An anticoagulant coagulopathy occurred in almost 80% of patients following rivaroxaban overdose, although bleeding was uncommon. There was a strong correlation between the INR and rivaroxaban concentrations, estimated by an anti‐factor Xa assay. This suggests that the INR can be used to monitor anticoagulation in rivaroxaban overdose. An INR ≤ 1.5 approximated therapeutic rivaroxaban concentrations and may prove an appropriate endpoint for observation. The median duration of rivaroxaban coagulopathy was 22 h and 94% of patients had an INR of ≤1.5 within 72 h.

Bleeding was uncommon despite anticoagulation occurring in most cases. A ceiling effect of factor Xa inhibition has been suggested to partly explain this finding following rivaroxaban overdose.^3^, ^6^ A volunteer dose‐escalation study of rivaroxaban in elderly subjects suggested a pharmacokinetic and pharmacodynamic ceiling effect at 50‐mg rivaroxaban,^10^ although the study did not assess doses higher than 50 mg. The two patients that developed significant bleeding in our series co‐ingested paracetamol and received acetylcysteine. Both paracetamol and acetylcysteine produce a functional factor VII deficiency, leading to prolongation of the prothrombin time.^11^, ^12^ It may be that additional disruption to the coagulation cascade and factor Xa inhibition puts a patient at higher risk of significant bleeding following rivaroxaban overdose. Neither patient with significant bleeding received factor replacement and the effectiveness of factor replacement to reverse coagulopathy following rivaroxaban overdose remains unclear. This possible increased risk of bleeding in rivaroxaban and paracetamol together in overdose, in the absence of paracetamol‐induced hepatotoxicity, may warrant further research, particularly because paracetamol was the second most common co‐ingested drug in this series. This is similar to a recognized interaction between warfarin and paracetamol.^13^

There was a strong correlation between INR and rivaroxaban concentrations estimated by an anti‐factor Xa assay in this series, suggesting an INR can be used to monitor patients following rivaroxaban overdose. Anti‐factor Xa assays were only performed in one fifth of cases reflecting their limited availability across smaller centres in Australia. In contrast, the INR is available at most locations. Performing serial INRs following rivaroxaban overdose to identify the peak INR and observing patients until the INR is ≤1.5 (approximating therapeutic rivaroxaban concentrations) appears reasonable and may prevent unnecessary transfer to a facility that can perform an anti‐factor Xa assay.

Co‐ingestions in this study were somewhat atypical when compared to other recent ATOM series in which benzodiazepine and antipsychotic co‐ingestions predominated.^14^, ^15^ Mirtazapine was co‐ingested most frequently in this series. This is likely explained in part by the population with access to rivaroxaban being an older cohort. The median age in this series of 49 years is higher than the median age of deliberate overdose in Australia of 32 years as reported in a large prospective series.^16^ In Australia, mirtazapine is a commonly prescribed antidepressant, particularly in the elderly population.^17^ Similarly, metoprolol was co‐ingested in almost 10% of presentations, likely reflecting those taking rivaroxaban often having cardiovascular comorbidities.

This study was limited by its observational nature with sampling times of coagulation studies determined by the treating clinician. It is possible that the assumed peak INR values do not represent true peaks, which may in part explain the lack of association detected between the reported rivaroxaban dose ingested and peak INR.

## 5 CONCLUSION

Bleeding was uncommon following rivaroxaban overdose. The INR appears to be a good indicator of coagulopathy, with an INR of ≤1.5 approximating therapeutic rivaroxaban concentrations. Observation of patients following acute rivaroxaban overdose until the INR ≤ 1.5 seems reasonable.

## AUTHOR CONTRIBUTIONS

Angela L. Chiew, Katherine Z. Isoardi and Geoffrey K. Isbister conceived the study. All authors were involved in patient recruitment and data collection. Katherine Z. Isoardi and Geoffrey K. Isbister performed data analysis. Katherine Z. Isoardi drafted the manuscript. All authors contributed to manuscript revision. Katherine Z. Isoardi takes responsibility for the paper as a whole.

## CONFLICT OF INTEREST STATEMENT

The authors declare no conflicts of interest.

## Supporting information


**Figure S1.** Preformatted datasheet
**Figure S2.** Graph demonstrating recruitment over time. The columns represent number of patients recruited annually (left y axis). The square points and line represent the annual median reported dose of rivaroxaban ingested (right y axis).
**Figure S3.** A scatter plot of the peak international normalized ratio (INR) following acute rivaroxaban overdose in groups that received charcoal and did not receive charcoal.

## Data Availability

The de‐identified data we analysed are not publicly available, but requests to the corresponding author will be considered on a case‐by‐case basis.
