# Supplementary material for: A prospective series of acute rivaroxaban overdose, coagulopathy and bleeding complications (ATOM 11)
Source: Br J Clin Pharmacol. 2026 Apr 24;92(8):2828–33. doi: 10.1002/bcp.70588 (PMC13420894; doi:10.1002/bcp.70588)

**Appendices**

Supplementary Figure 1: Preformatted datasheet


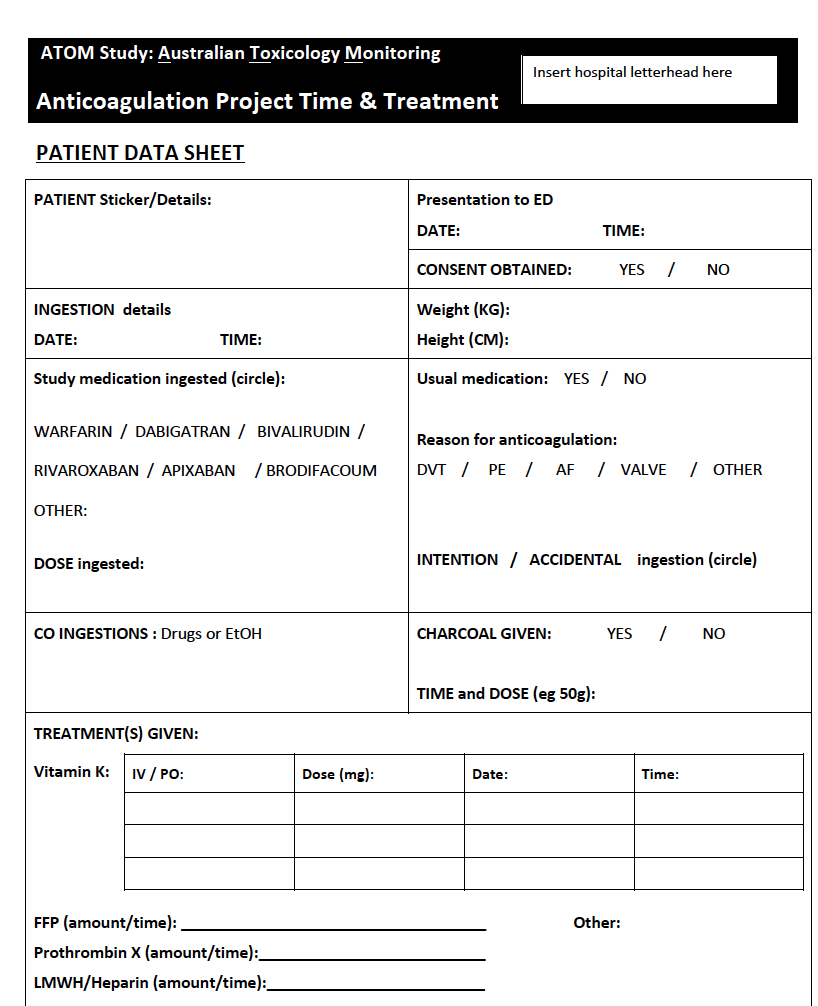


Supplementary Figure 2: Graph demonstrating recruitment over time. The columns represent number of patients recruited annually (left y axis). The square points and line represent the annual median reported dose of rivaroxaban ingested (right y axis).


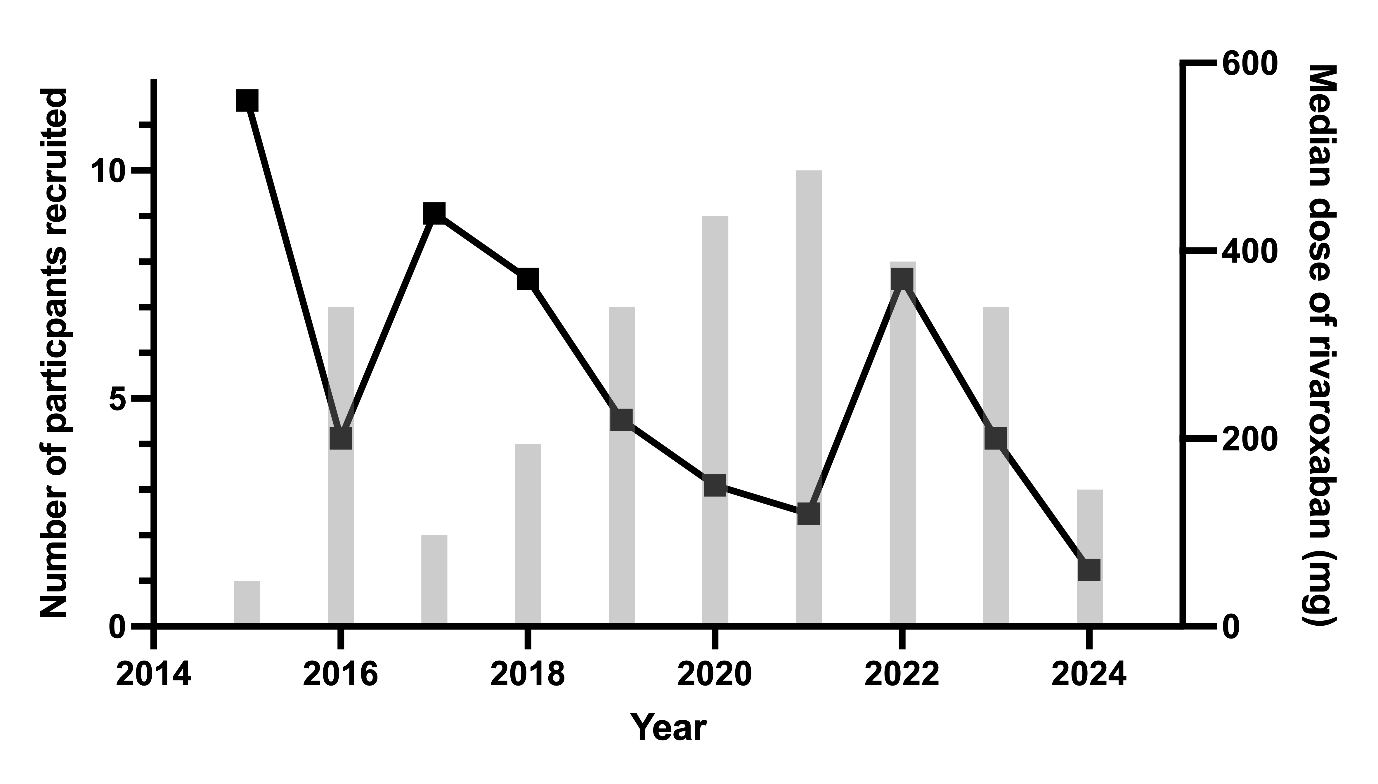


Supplementary Figure 3: A scatter plot of the peak international normalised ratio (INR) following acute rivaroxaban overdose in groups that received charcoal and did not receive charcoal.


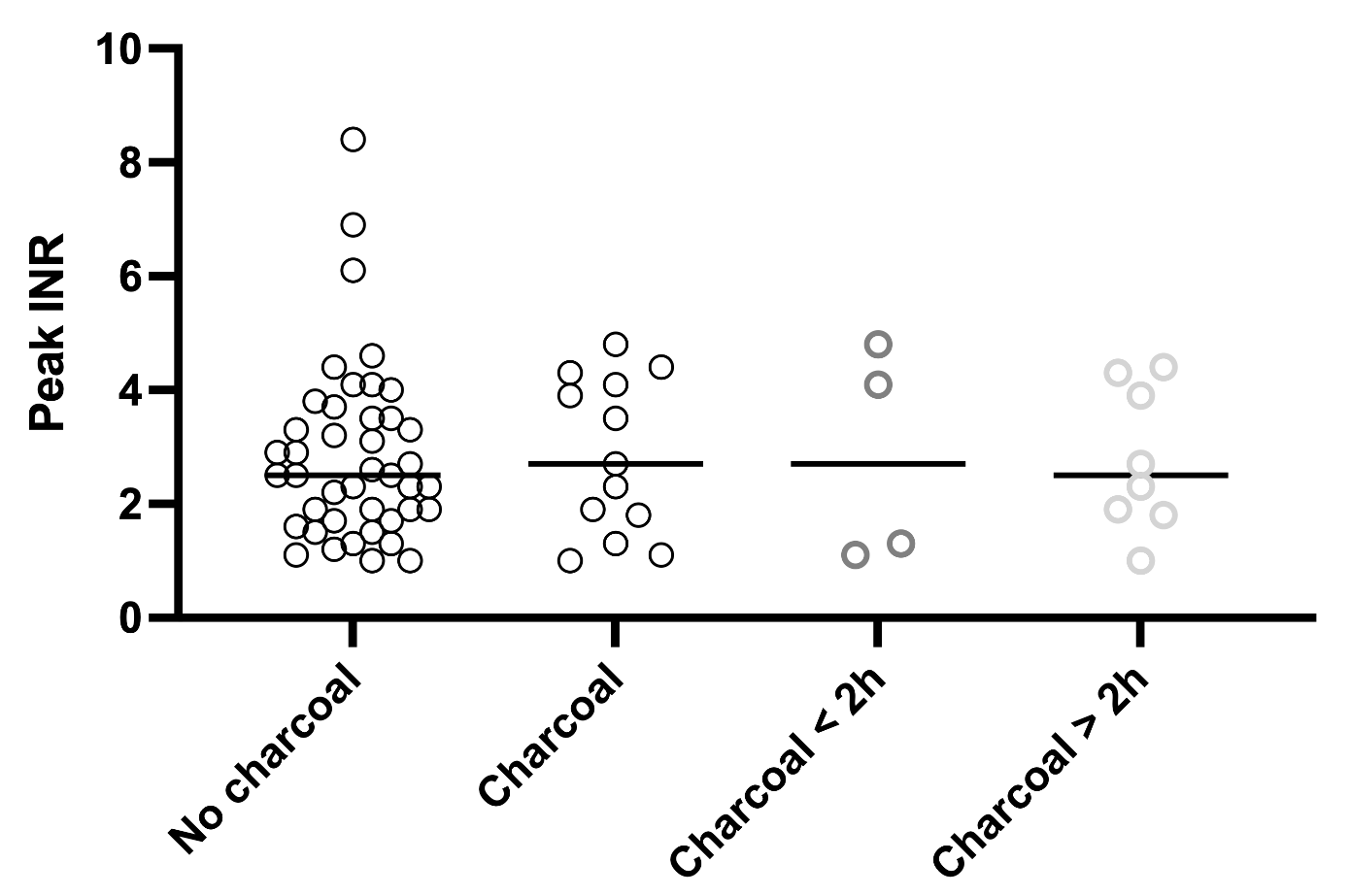

Supplement: Supplementary file 1 — Figure S1. Preformatted datasheet Figure S2. Graph demonstrating recruitment over time. The columns represent number of patients recruited annually (left y axis). The square points and line represent the annual median reported dose of rivaroxaban ingested (right y axis). Figure S3. A scatter plot of the peak international normalized ratio (INR) following acute rivaroxaban overdose in groups that received charcoal and did not receive charcoal. [file BCP-92-2828-s001.docx]
